# Bilateral Granulomatous Keratic Precipitates Following Inadequate Antibiotic Coverage for Presumed Lyme Disease

**DOI:** 10.7759/cureus.95917

**Published:** 2025-11-01

**Authors:** Michael J Miyashiro, Nikki P Inamine, Gary S Inamine

**Affiliations:** 1 Ophthalmology, Eyeball Doc LLC, Bradenton, USA; 2 Internal Medicine, The Queen's Health Systems, Honolulu, USA

**Keywords:** anterior uveitis, borrelia burgdorferi, case report, corneal endothelium, granulomatous uveitis, interstitial keratitis, keratic precipitates, lyme disease, ocular lyme disease, tick-borne infection

## Abstract

Lyme disease, caused by *Borrelia burgdorferi*, is a multisystem infection with diverse ocular manifestations. Although ocular involvement is uncommon, it can include conjunctivitis, keratitis, uveitis, and optic neuropathy.

We describe a 61-year-old man who developed bilateral mutton-fat keratic precipitates following a tick bite and incomplete systemic antibiotic coverage. His course was complicated by premature discontinuation of doxycycline and systemic corticosteroid exposure. Ophthalmic examination revealed bilateral granulomatous keratic precipitates without anterior chamber reaction. He was prescribed systemic azithromycin and referred to an infectious disease specialist for further evaluation. At the one-month follow-up, the patient demonstrated complete resolution of keratic precipitates and anterior chamber inflammation after completing azithromycin therapy, without the need for adjunctive corticosteroid use.

This case underscores the importance of considering Lyme disease in the differential diagnosis of bilateral granulomatous keratic precipitates. Diagnostic challenges included an early false-negative serologic result, premature discontinuation of antibiotics, and corticosteroid exposure without adequate antimicrobial coverage.

Bilateral granulomatous keratic precipitates may represent an early ophthalmic marker of systemic Lyme disease. Prompt recognition, initiation of systemic antibiotic therapy, and referral to an infectious disease specialist are essential to prevent long-term sequelae.

## Introduction

Lyme disease is the most common vector-borne illness in North America and Europe, transmitted to humans primarily through the bite of Ixodes ticks carrying *Borrelia burgdorferi* [1]. Since its initial description in Lyme, Connecticut, in the mid-1970s, the incidence of Lyme disease has continued to rise, with tens of thousands of cases reported annually in the United States and additional cases across Europe. The disease follows a multisystemic course that may involve dermatologic, musculoskeletal, neurologic, cardiac, and ocular systems.

Ocular manifestations of Lyme disease are relatively uncommon compared with systemic features but are clinically important because they can mimic other infectious and inflammatory conditions. The reported spectrum of ocular involvement ranges from mild conjunctivitis and episcleritis to keratitis, anterior and intermediate uveitis, retinal vasculitis, and optic neuropathy [2-4]. This diversity often complicates diagnosis, particularly when serologic confirmation is absent or delayed. Ocular involvement accounts for fewer than 5% of all Lyme disease cases, and bilateral granulomatous keratic precipitates are exceedingly rare, primarily described in isolated case reports.

Granulomatous keratic precipitates are an especially uncommon finding in Lyme-associated uveitis. While isolated reports have described keratitis and interstitial keratitis in Lyme disease, bilateral granulomatous keratic precipitates remain rare and may be mistaken for other infectious or autoimmune etiologies [2-4]. Recognizing this presentation is critical, as inadequate or incomplete antimicrobial therapy may allow disease progression, potentially worsened by corticosteroid exposure without appropriate antibiotic coverage. Clinicians should maintain a high index of suspicion in endemic regions and confirm infectious clearance before initiating corticosteroid treatment.

Here, we describe a case of presumed Lyme disease in which a patient developed bilateral granulomatous keratic precipitates following premature discontinuation of doxycycline and systemic corticosteroid use. This case underscores the importance of maintaining clinical suspicion for ocular Lyme disease, ensuring adequate systemic antibiotic therapy, and pursuing timely multidisciplinary evaluation.

## Case presentation

A 61-year-old Caucasian man reported a tick bite on his torso in July 2025. Three weeks later, he developed systemic malaise and myalgias. He was treated at an urgent care center with intramuscular corticosteroids and oral doxycycline, which he discontinued after five doses due to headache. His systemic symptoms relapsed, and by late August, he developed bilateral eye redness, photophobia, and blurred vision.

At ophthalmic evaluation, best-corrected visual acuity was 20/40 in the right eye (OD) and 20/25 in the left eye (OS). Intraocular pressures were 14 mmHg in both eyes. Pupils, visual fields, and ocular motility were normal.

**Figure 1:**
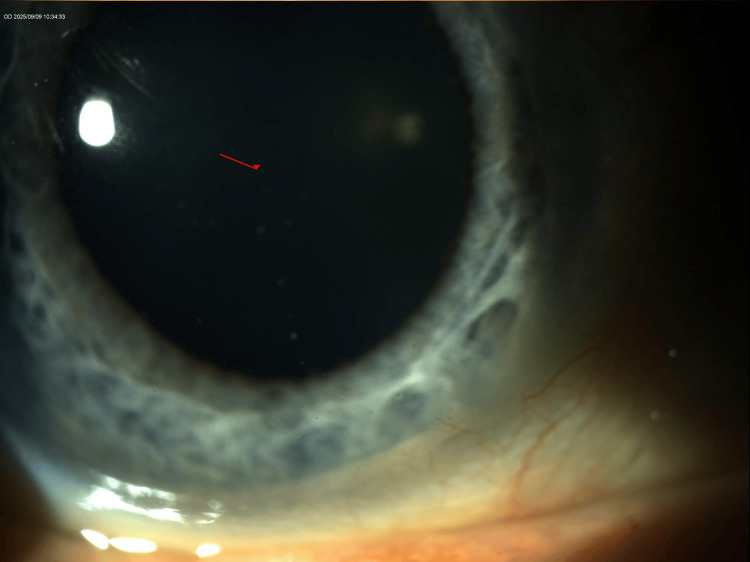
Annotated slit-lamp photo of the right eye (OD) demonstrating multiple mutton-fat keratic precipitates on the corneal endothelium.

**Figure 2:**
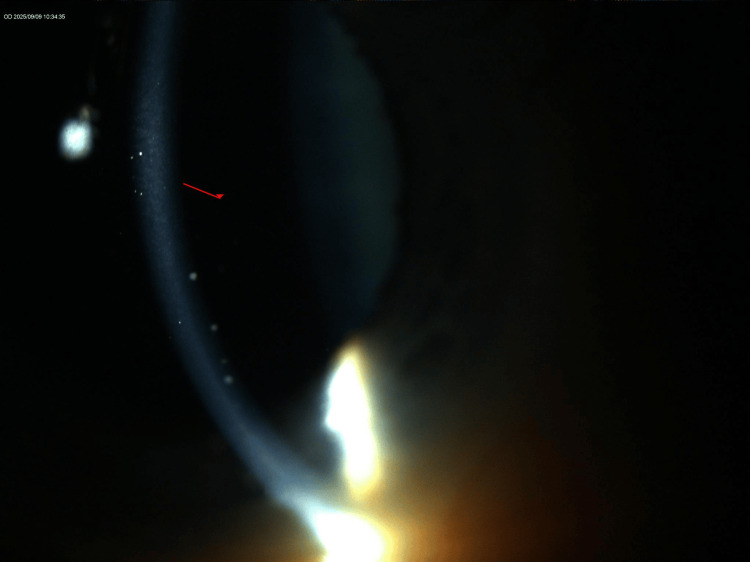
Narrow-beam slit-lamp image of the right eye (OD) highlighting anterior chamber reaction with keratic precipitates.

**Figure 3:**
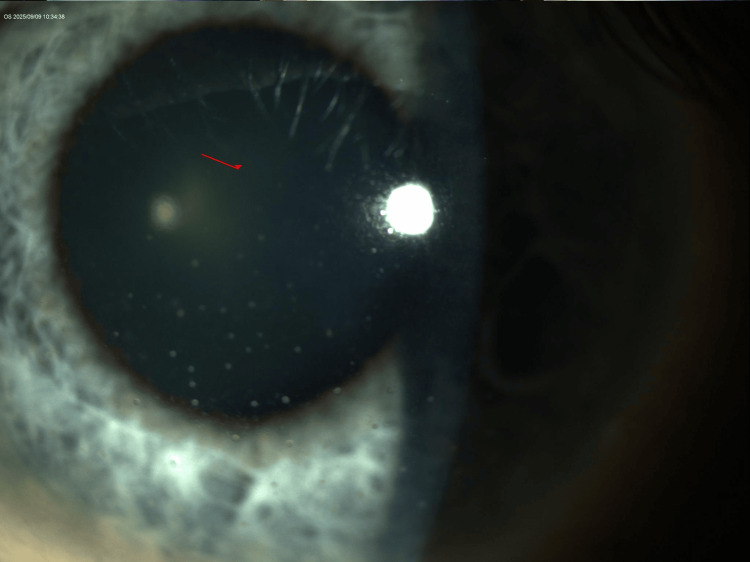
Annotated slit-lamp photo of the left eye (OS) showing diffuse distribution of granulomatous keratic precipitates.

**Figure 4:**
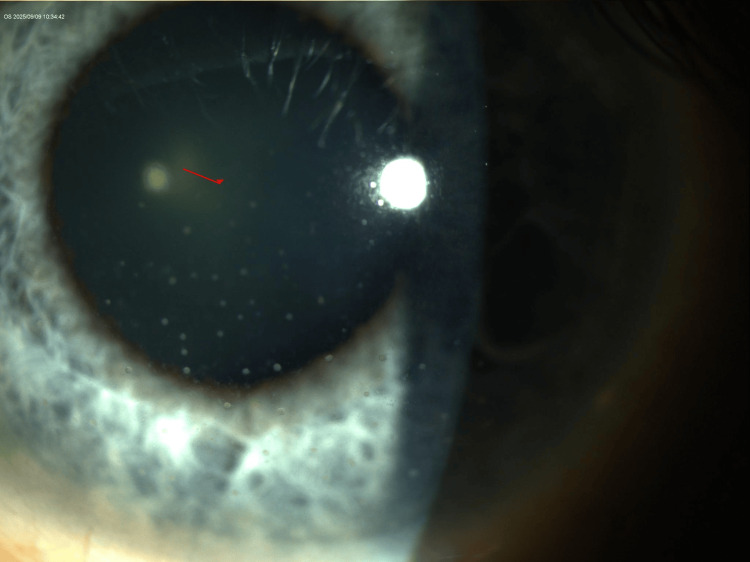
Higher magnification slit-lamp photograph of the left eye (OS) demonstrating multiple large mutton-fat keratic precipitates clustered on the corneal endothelium. The anterior chamber remains quiet without evidence of flare or hypopyon.

Slit-lamp examination revealed bilateral mutton-fat keratic precipitates, more prominent in the OS, with otherwise quiet anterior chambers and clear vitreous (Figures 1-4). The lenses showed trace nuclear sclerosis bilaterally. Posterior segment examination was unremarkable in both eyes; therefore, fundus imaging was not performed.

The diagnosis of Lyme disease was presumed based on the patient’s recent tick exposure, systemic symptoms, and subsequent development of bilateral granulomatous keratic precipitates. Confirmatory serologic testing was not yet available at the time of this report, and the patient was referred to an infectious disease specialist for repeat testing and systemic evaluation. At the time of submission, confirmatory testing and consultation were pending; post-submission findings were not used to infer causation.

The patient was prescribed azithromycin 500 mg three times daily for 21 days as an alternative regimen due to doxycycline intolerance. Azithromycin was selected in accordance with the 2020 IDSA/AAN/ACR guidelines as a second-line agent for patients unable to tolerate first-line therapies (doxycycline, amoxicillin, or cefuroxime).

## Discussion

This case illustrates several important clinical considerations previously described in the context of ocular Lyme disease [2-4]. First, premature discontinuation of doxycycline may allow relapse or progression of systemic and ocular manifestations. Second, the administration of systemic corticosteroids without concurrent antibiotic therapy may worsen disease progression. Finally, bilateral granulomatous keratic precipitates, although rare, represent a clinically significant ocular manifestation of presumed Lyme disease. Ocular Lyme disease remains a diagnostic challenge; serologic testing may be negative early in infection, particularly after prior antibiotic or corticosteroid exposure. A comprehensive systemic evaluation, including serology, imaging, and exclusion of alternative causes such as sarcoidosis, syphilis, herpes simplex virus, varicella-zoster virus, and autoimmune disease, is essential to confirm the etiology.

The management of this patient also highlights the role of alternative antibiotic therapy. Although azithromycin is less commonly used as first-line treatment, it may serve as a useful option for patients who cannot tolerate doxycycline. Prompt referral to infectious disease specialists ensures thorough systemic evaluation, serologic confirmation, and multidisciplinary follow-up. A limitation of this report is that confirmatory Lyme serologic testing was pending at the time of publication, as the patient had not yet been evaluated by an infectious disease specialist for formal testing and follow-up. At one-month follow-up, the patient demonstrated complete resolution of ocular inflammation after completing azithromycin therapy, without adjunctive corticosteroid use.

## Conclusions

Bilateral keratic precipitates, particularly of the granulomatous type, should raise suspicion for infectious etiologies, including Lyme disease, especially in endemic regions. Adequate systemic antibiotic therapy and coordinated care between ophthalmology and infectious diseases are essential to preventing vision-threatening complications. This case represents a presumed Lyme-associated uveitis limited by a lack of confirmatory serology at the time of writing. It underscores the need to verify infectious resolution prior to corticosteroid initiation and the importance of interdisciplinary follow-up for diagnostic confirmation.
